# Antioxidant and Neuroprotective Effects Induced by Cannabidiol and Cannabigerol in Rat CTX-TNA2 Astrocytes and Isolated Cortexes

**DOI:** 10.3390/ijms21103575

**Published:** 2020-05-18

**Authors:** Viviana di Giacomo, Annalisa Chiavaroli, Lucia Recinella, Giustino Orlando, Amelia Cataldi, Monica Rapino, Valentina Di Valerio, Maurizio Ronci, Sheila Leone, Luigi Brunetti, Luigi Menghini, Gokhan Zengin, Gunes Ak, Hassan H. Abdallah, Claudio Ferrante

**Affiliations:** 1 Department of Pharmacy, Università degli Studi “Gabriele d’Annunzio”, via dei Vestini 31, 66100 Chieti, Italy; viviana.digiacomo@unich.it (V.d.G.); annalisa.chiavaroli@unich.it (A.C.); lucia.recinella@unich.it (L.R.); amelia.cataldi@unich.it (A.C.); maurizio.ronci@unich.it (M.R.); sheila.leone@unich.it (S.L.); luigi.brunetti@unich.it (L.B.); luigi.menghini@unich.it (L.M.); claudio.ferrante@unich.it (C.F.); 2 Genetic Molecular Institute of CNR, Unit of Chieti, “G. d’ Annunzio” University, Via dei Vestini 31, 66100 Chieti-Pescara, Italy; m.rapino@unich.it; 3 Department of Medicine and Ageing Sciences, “G. d’ Annunzio” University, Via dei Vestini 31, 66100 Chieti-Pescara, Italy; valentina.divalerio@unich.it; 4 Department of Biology, Science Faculty, Selcuk University, Campus, 42130 Konya, Turkey; akguneselcuk@gmail.com; 5 Chemistry Department, College of Education, Salahaddin University-Erbil, Erbil 44001, Iraq; Hassan.Abdullah@su.edu.krd; 6 School of Pharmaceutical Sciences, University Sains Malaysia, USM, Penang 11800, Malaysia

**Keywords:** cannabidiol, cannabigerol, neuroprotection, serotonin, apoptosis, proteomic analysis, oxidative stress, exocytosis, docking

## Abstract

Cannabidiol (CBD) and cannabigerol (CBG) are *Cannabis sativa* terpenophenols. Although CBD’s effectiveness against neurological diseases has already been demonstrated, nothing is known about CBG. Therefore, a comparison of the effects of these compounds was performed in two experimental models mimicking the oxidative stress and neurotoxicity occurring in neurological diseases. Rat astrocytes were exposed to hydrogen peroxide and cell viability, reactive oxygen species production and apoptosis occurrence were investigated. Cortexes were exposed to K^+^ 60 mM depolarizing stimulus and serotonin (5-HT) turnover, 3-hydroxykinurenine and kynurenic acid levels were measured. A proteomic analysis and bioinformatics and docking studies were performed. Both compounds exerted antioxidant effects in astrocytes and restored the cortex level of 5-HT depleted by neurotoxic stimuli, whereas sole CBD restored the basal levels of 3-hydroxykinurenine and kynurenic acid. CBG was less effective than CBD in restoring the levels of proteins involved in neurotransmitter exocytosis. Docking analyses predicted the inhibitory effects of these compounds towards the neurokinin B receptor. *Conclusion:* The results in the in vitro system suggest brain non-neuronal cells as a target in the treatment of oxidative conditions, whereas findings in the ex vivo system and docking analyses imply the potential roles of CBD and CBG as neuroprotective agents.

## 1. Introduction

*Cannabis sativa* belongs to the *Cannabaceae* family and is classified in the three recognized varieties *sativa*, *indica* and *ruderalis.* It is a medicinal plant characterized by a phytocomplex rich in secondary metabolites (more than 500), with at least 100 terpenophenolic compounds [1]. The Δ9-tetrahydrocannabinol (THC) has long been considered the sole psychotropic molecule, but a novel phytocannabinoid, the Δ9-tetrahydrocannabiphorol, which is even more potent than THC itself, was recently identified in *C. sativa* phytocomplex [2]. Cannabidiol (CBD) is another terpenophenol that has long been studied from a pharma-toxicological point of view [3,4,5,6]. Despite having a chemical structure very close to that of the THC, CBD was reported to act with a different multitarget mechanism, including the binding to more than 60 receptor proteins. Among the others, the activation of the 5-hydroxytryptamine receptor 1A (5-HT1A) is considered as one of the main mechanisms underlying the neuroprotective effects of CBD in neurological diseases [7,8,9]. Cannabigerol (CBG), another *C. sativa* terpenophenol which shares a similar mechanism of action with CBD, was also reported to act as a neuroprotective agent and to bind to the 5-HT1A receptor. CBG use in treating neurological diseases is, however, still unexplored [10]. In a very recent paper, the treatment of rat hypothalamus with CBD resulted in an increase in the basal 5-HT level, and a similar trend was found for CBG [11]. Nevertheless, the effect of these two compounds on brain 5-HT synthesis and release, in the framework of pro-oxidant/pro-inflammatory conditions underlying neurological disorders, needs to be better elucidated.

As the most abundant cell type in the brain, the astrocytes have gained enormous interest in recent decades as a potential target for neurotherapies, due to their essential and pleiotropic roles in brain physiology and pathology. They provide critical metabolic and structural support to neurons [12] and their metabolic coordination with neurons is crucial to maintain brain bioenergetic homeostasis and to combat threats such as oxidative stress [13]. Oxidative stress is involved in the pathophysiology of numerous diseases of the nervous system. Of all brain cell types, neurons are particularly susceptible to redox changes due to their high metabolic rate and limited antioxidant capacity [14]. Astrocytes, in contrast, have greater antioxidative potential [15]. Multiple studies have demonstrated that the astrocytic support of the neuronal antioxidant system is a key neuroprotective mechanism against oxidative damage [16]. Although we still have a limited understanding of the mechanisms of CBD neuroprotection, it is clear by now that the drug’s effects are not limited to the direct modulation of neurons, but are extended to microglia and astrocytes [17]. In different models of brain ischemia and/or neurogenesis, the positive effects exerted by CBD are strongly related to the astrocyte response to the compound [18,19]. Cannabigerol and its derivatives in neuronal diseases are actually less studied than their counterparts [20], although CBG efficacy in reducing oxidative stress and apoptosis was demonstrated in a model of neuroinflammation [21].

The present study aims therefore to investigate and compare the antioxidant and neuroprotective role of CBD and CBG in two experimental models. In the in vitro model, a rat cell line of astrocytes, which could represent a key target for neurotherapies [22], was exposed to an oxidative stimulus (hydrogen peroxide) and the effects of CBD and CBG were evaluated in terms of cell viability, reactive oxygen species (ROS) production and apoptosis occurrence. In parallel, in the ex vivo model, isolated rat cortexes were exposed to the same concentrations of hydrogen peroxide and of the two terpenophenols, and nitrite production and serotonin (5-HT) concentration were assessed. In addition, a supra-physiological depolarizing stimulus (K^+^ 60 mM) [22] was administered to the cortexes in order to reproduce the pathophysiological conditions that are common in various neurological diseases, namely migraine, hypoxia/ischemia and epilepsy [23]. Following treatment with both CBD and CBG, the turnover of 5-HT was determined alongside proteomic analysis and bioinformatics and docking studies. These analyses aimed to investigate the possible mechanisms underlying the effects of the two compounds on cell survival and exocytosis processes leading to 5-HT release. 

## 2. Results

### 2.1. Antioxidant Activity

First, cannabidiol (CBD) and cannabigerol (CBG) were assayed to investigate their intrinsic scavenging/reducing activity and enzyme inhibition properties through colorimetric assays. The results depicted in Table 1 showed similar IC_50_ values in their anti-cholinesterase effects. The IC_50_ values were much higher (1.04–1.88 mM) compared to that displayed by the reference enzyme inhibitor, galantamine (IC_50_: 0.01–0.02 mM). Any pharmacological relevance of the anti-cholinesterase activity displayed by both CBD and CBG can therefore be excluded, considering as well that most of the relevant interactions with putative targets occur at concentrations in the submicromolar–micromolar range.

In contrast, their antiradical effects were almost identical in three of the scavenging/reducing tests: the 2,2-diphenyl-1-picrylhydrazy (DPPH), the 2,2′-azino-bis (3-ethylbenzothiazoline-6-sulfonic acid (ABTS) and the ferric reducing antioxidant power (FRAP) tests (Table 2). Both CBD and CBG have IC_50_ values higher than the reference compound, Trolox. On the other hand, in the cupric reducing antioxidant capacity (CUPRAC) test, CBG showed a higher reducing power than CBD, with an IC_50_ value (0.59 ± 0.01) very similar to that of Trolox (0.50 ± 0.01).

### 2.2. In Vitro Model

#### 2.2.1. MTT Assay

**Figure 1:**
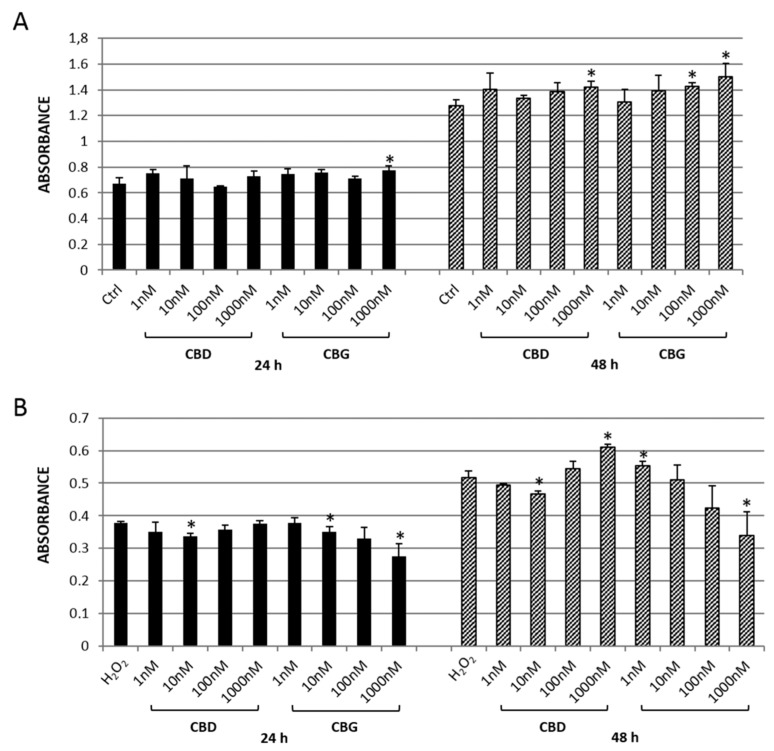
MTT assay of CTX-TNA2 astrocyte cell line exposed to different concentrations (1–1000 nM) of either cannabidiol (CBD) or cannabigerol (CBG) for 24 (black bars) and 48 h (grey bars). (**A**) Cells in basal conditions. (**B**) Cells challenged with 300 µM H_2_O_2_. The data graph bars are the mean ± SD (*n* = 3). ANOVA, * *p* < 0.05 vs. control group.

Once the antioxidant potential of CBD and CBG was assessed, they were tested on the CTX-TNA2 rat astrocyte cell line at different concentrations (range 1–1000 nM) (Figure 1). The astrocytes cultured in basal conditions and exposed to the highest concentrations of both cannabidiol (1000 nM) and cannabigerol (100 and 1000 nM) showed an increase in cell proliferation after 48 h of treatment (Figure 1A). The same concentration of CBD proved effective in protecting the cells from oxidative damage exerted by the exposure to H_2_O_2_. In contrast, CBG was effective at the lowest concentration (1 nM) (Figure 1B). Both compounds were found to be effective, but at different concentrations, in counteracting the cytotoxicity that occurred after the exposure to H_2_O_2_.

#### 2.2.2. Reactive Oxygen Species (ROS) Production

**Figure 2:**
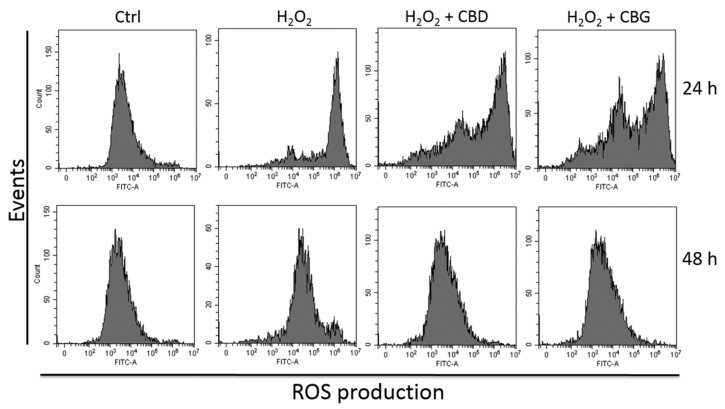
Reactive oxygen species (ROS) production in CTX-TNA2 astrocyte cell line exposed to 1000 nM CBD and 1 nM CBG and challenged with H_2_O_2_ for 24 and 48 h. One representative experiment out of three independent experiments is shown (*n* = 3).

Based on the previous results, the highest concentration of CBD (1000 nM) and the lowest concentration for CBG (1 nM) were chosen for further analyses aimed at investigating their antioxidant potential. Consequently, the ROS production was analyzed in CTX-TNA2 cells (Figure 2). An effect in reverting the ROS production triggered by H_2_O_2_ was observed after 24 h of treatment with both terpenophenols. In addition, the reduction in ROS production, in the presence of both CBD and CBG, with respect to the sample exposed to 300 µM H_2_O_2_ only, was observed after 48 h as well. The same concentrations able to reduce the H_2_O_2_-induced cytotoxicity after 48 h, showed a reduction in ROS production already present after 24 h of treatment and lasting up to 48 h.

#### 2.2.3. Apoptosis Occurrence

**Figure 3:**
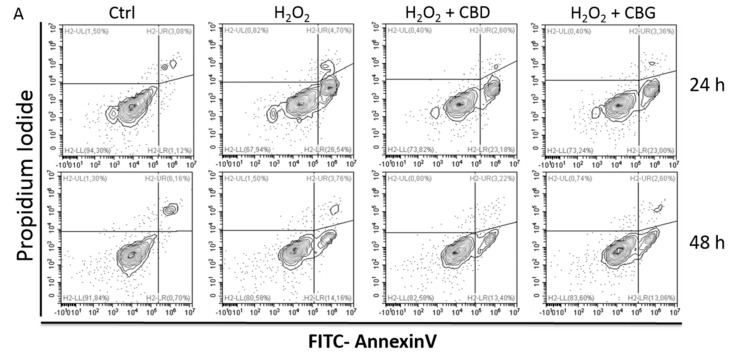

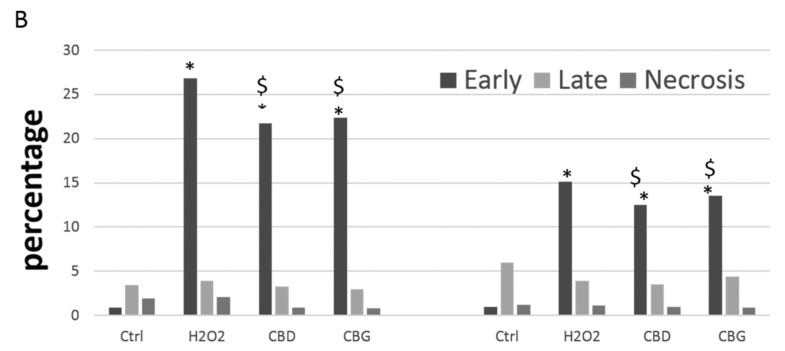
Apoptosis occurrence in CTX-TNA2 astrocyte cell line exposed to 1000 nM CBD and 1 nM CBG and challenged with H_2_O_2_ for 24 and 48 h. ANOVA, * *p* < 0.05 vs. control group; $ *p* < 0.05 vs. H_2_O_2_ group. (**A**) A representative experiment (**B**) The graph shows the mean ± SD (*n* = 3). Early: early apoptosis, i.e., Annexin-V^pos^ and PI^neg^ cells Late: late apoptosis, i.e., Annexin-V^pos^ and PI^pos^ cells. Necrosis: Annexin-V^neg^ and PI^pos^ cells.

The occurrence of apoptosis was evaluated as a possible effect of oxidative stress (Figure 3). Interestingly, the two compounds, CBD 1000 nM and CBG 1 nM, were able to reduce early apoptosis (represented by Annexin-V^pos^/PI^neg^ cells) after 24 h and the effect lasted up to 48 h.

#### 2.2.4. Western Blotting Analysis

**Figure 4:**
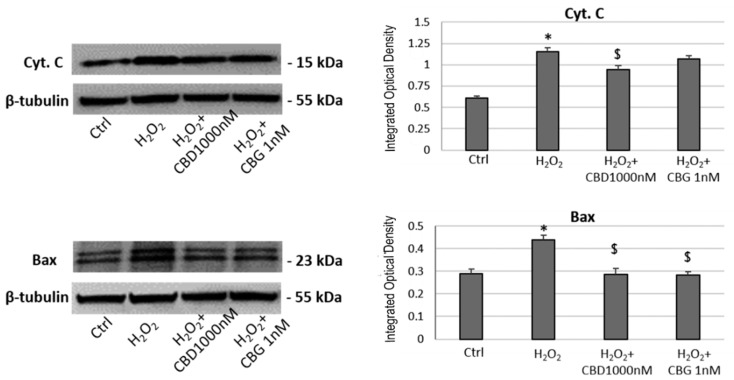
Western blotting analysis in CTX-TNA2 astrocyte cell line exposed to 1000 nM CBD and 1 nM CBG and challenged with H_2_O_2_ for 24 h. The graph shows the mean ± SD (*n* = 3). ANOVA, * *p* < 0.05 vs. Ctrl group; $ *p* < 0.05 vs. H_2_O_2_ group. A representative western blotting for each protein is shown.

The analysis of apoptosis-related protein expression showed an increase in both Bax and cytochrome C (Cyt C) expression in the H_2_O_2_-treated cells with respect to the control sample. CBD was able to keep Bax expression at values comparable to the control sample and to decrease the Cyt C levels in comparison to H_2_O_2_ sample. CBG had effect on Bax only, whose expression does not differ from the control sample (Figure 4). In line with the previous assay, CBD was able to keep the level of the two proteins related to apoptosis at values similar to the control sample. CBG, on the other hand, was effective only on the Bax level.

### 2.3. Ex Vivo Studies

#### 2.3.1. Oxidative Stress and Nitrite Production and Serotonin (5-HT) Concentration

**Figure 5:**
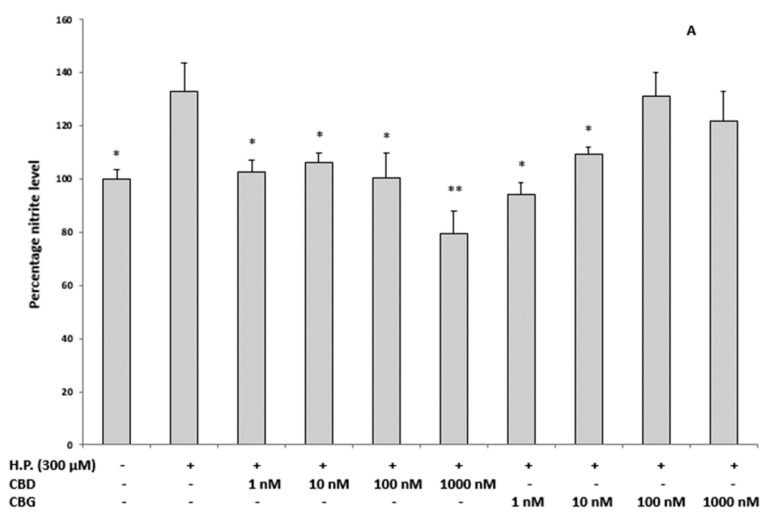

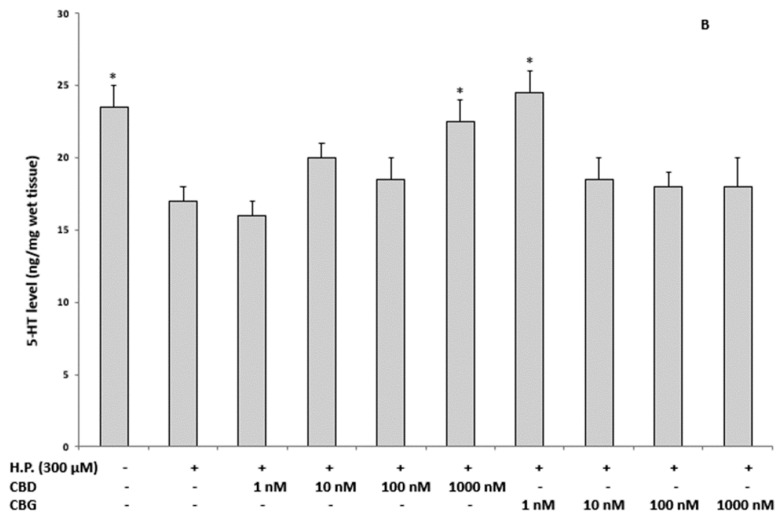
Inhibitory effects of CBD and CBG 1–1000 nM on H_2_O_2_-induced increase in nitrite level (expressed as percentage level compared to control group; panel (**A**) and reduction in 5-HT level (expressed as ng/mg wet tissue; panel (**B**), in isolated rat cortexes (*n* = 3 for each experimental condition). Panel A: ANOVA, *p* < 0.001, F = 14.02; *post hoc* test, * *p* < 0.05, ** *p* < 0.01 vs. hydrogen peroxide (H.P.) group. Panel B: ANOVA, *p* < 0.001, F = 12.99; *post hoc* test, * *p* < 0.05 vs. hydrogen peroxide (H.P.) group.

On the basis of the results obtained in the in vitro model, the antioxidant potential of CBD and CBG was tested in the ex vivo model as well. In this model, isolated prefrontal cortexes from rats were exposed to hydrogen peroxide 300 µM and to all the concentrations (ranging 1–1000 nM) of CBD and CBG, in the search for the best concentration in this varied experimental system. The exposure to H_2_O_2_ increased the nitrite production with respect to the control sample. CBD was able to inhibit such an increase at all the tested concentrations, whereas the inhibitory effect exerted by CBG was significant at the two lowest tested concentrations only. The maximal CBG inhibition is registered for the 1 nM concentration (Figure 5A).

The 5-HT concentration was determined in the same experimental conditions and found to be higher in the control sample with respect to the cortexes exposed to H_2_O_2_. CBD showed a blunting effect on 5-HT depletion induced by H_2_O_2_ when administered at 1000 nM, while CBG has the same effect at the concentration of 1 nM (Figure 5B). The exposure of cortexes to H_2_O_2_ induces nitrite production, an index of cytotoxicity, and decrease the concentration of the physiological neurotransmitter 5-HT. All CBD concentrations were effective in counteracting the nitrite production, whereas only the highest concentration (1000 nM) restored the 5-HT levels. On the other hand, CBG showed the best result in both assays when administered at the lowest concentration (1 nM).

#### 2.3.2. Neurotoxic Stimulus and 3-Hydrokinurenine (3-HK) and Kynurenic acid (KA) Production, and 5-HT Turnover

**Figure 6:**
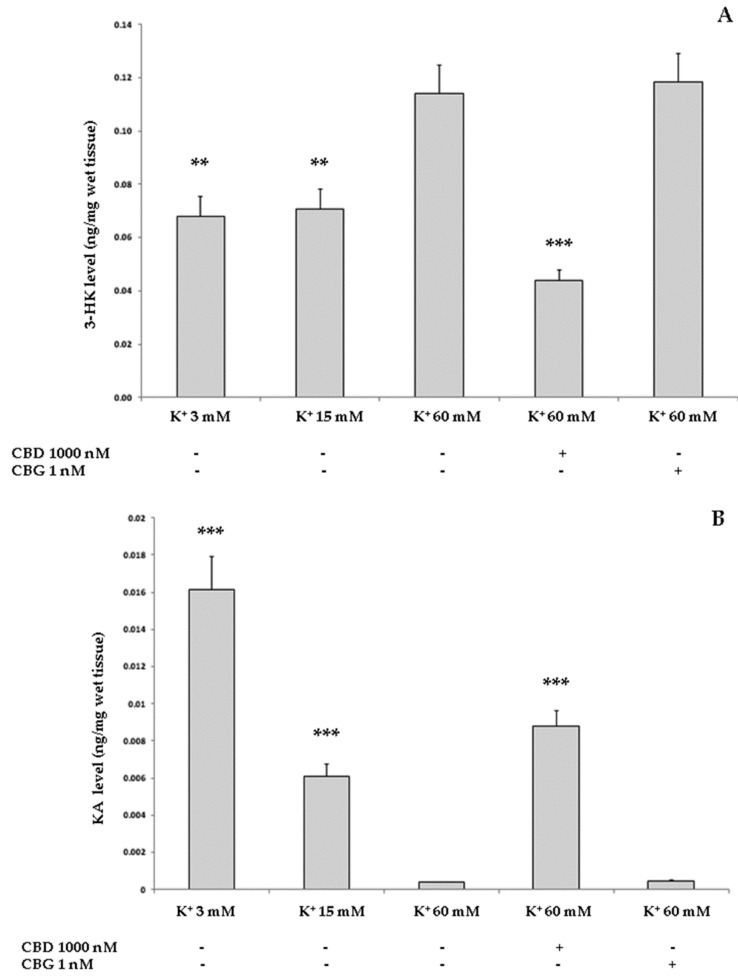

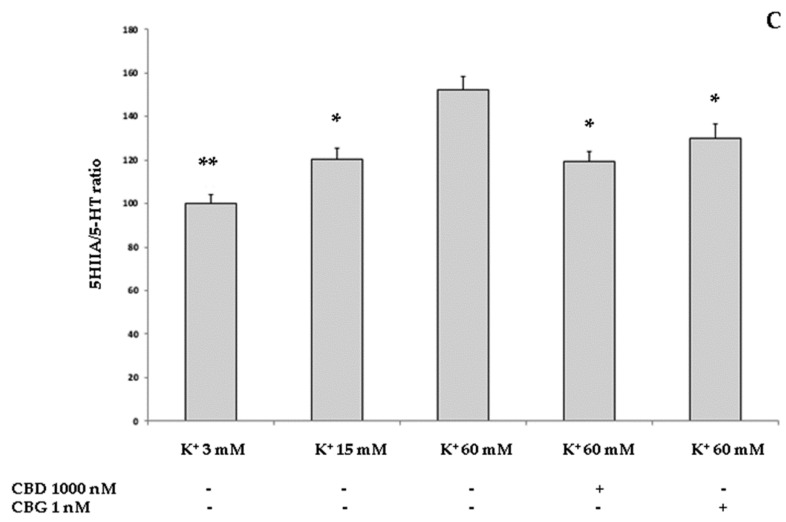
Effects of CBD 1000 nM and CBG 1 nM on K^+^ 60 mM-induced increase in 3-HK level (expressed as ng/mg wet tissue; Panel (**A**), reduction in kynurenic acid (KA) level (expressed as ng/mg wet tissue; Panel (**B**), and increase in 5-HT turnover (measured as 5HIIA/5-HT ratio, Panel **C**) in isolated rat cortexes (*n* = 3 for each experimental condition). Panel A: ANOVA, *p* < 0.001, F = 41.26; *post hoc* test, ** *p* < 0.01, *** *p* < 0.001 vs. K^+^ 60 mM group. Panel B: ANOVA, *p* < 0.001, F = 91.51; *post hoc* test, *** *p* < 0.001 vs. K^+^ 60 mM group. Panel C: ANOVA, *p* < 0.001, F = 32.37; *post hoc* test, * *p* < 0.05, ** *p* < 0.01 vs. K^+^ 60 mM group.

Considering these findings, two concentrations, namely 1000 nM for CBD and 1 nM for CBG, were chosen to perform further pharmacological tests. In a second set of ex vivo experiments, the isolated rat cortexes were exposed to a neurotoxic stimulus represented by an external K^+^ concentration of 60 mM, corresponding to an excitotoxic depolarizing stimulus. The control samples were exposed to the K^+^ concentrations of 3 mM and 15 mM, representing basal and physiological depolarizing stimuli, respectively. In these experimental conditions, the levels of 3-HK and kynurenic acid (KA) were measured, with the first being a well-known index of neurotoxicity and the second a marker of neuroprotection [24]. Compared to the two physiological conditions, the neurotoxic stimulus upregulated the level of 3-HK and downregulated the KA concentration. If CBD 1000 nM proved successful in blunting K^+^ 60 mM-induced alterations of both 3-HK and KA levels, CBG 1 nM was completely ineffective (Figure 6A,B).

In the same experimental conditions, the K^+^ 60 mM stimulus significantly increased the 5-HT turnover in rat cortexes, measured as the 5-hydroxyindolacetic/serotonin (5HIIA/5-HT) ratio. Both CBD 1000 nM and CBG 1 nM were able to prevent the 5-HT depletion induced by the neurotoxic stimulus (Figure 6C). CBD was effective in reverting all the changes induced by the neurotoxic stimulus, represented by the exposure to a K^+^ concentration of 60 mM. Conversely, CBG succeeded only in decreasing the 5-HT turnover. 

#### 2.3.3. Neurotoxic Stimulus and Proteomic Analysis

**Figure 7:**
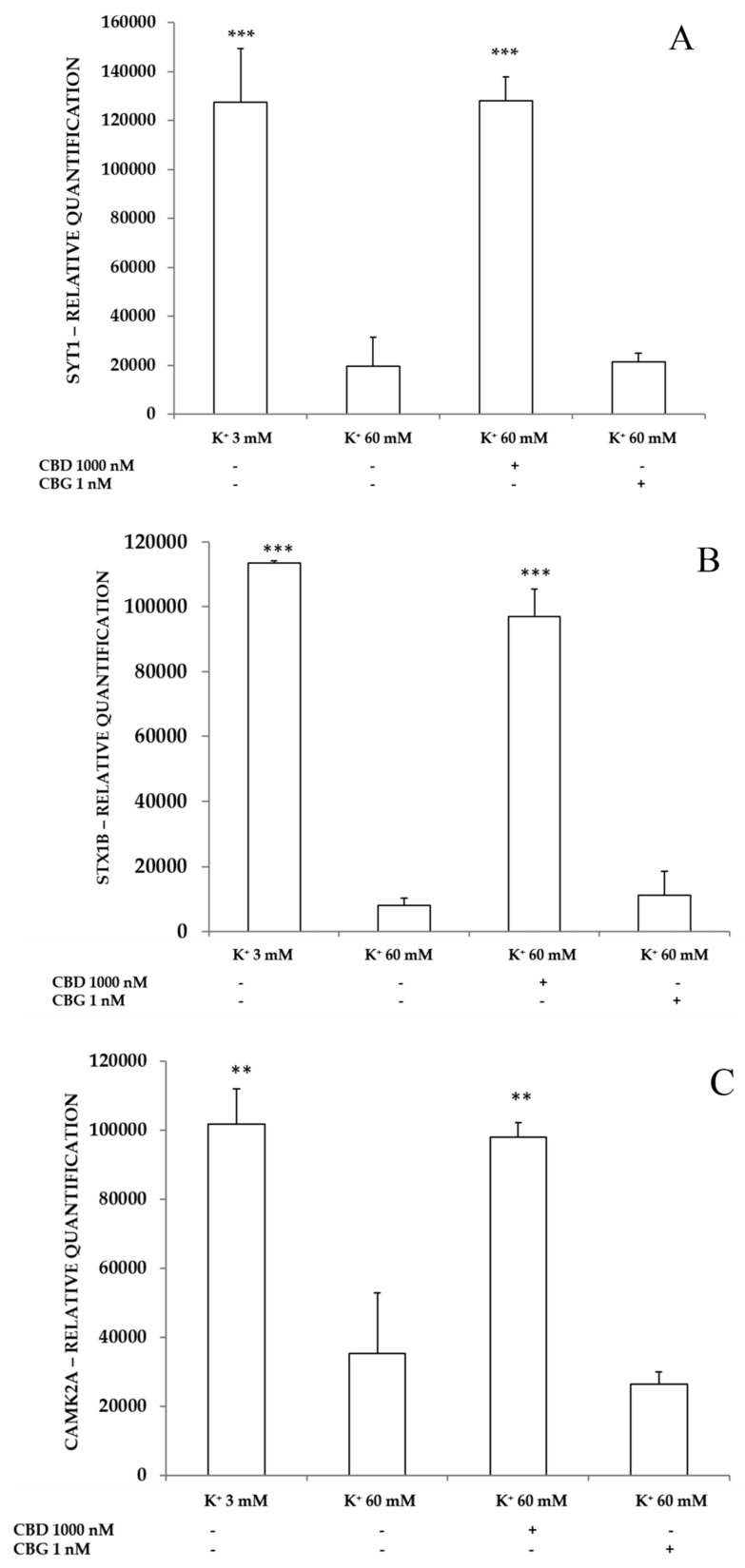

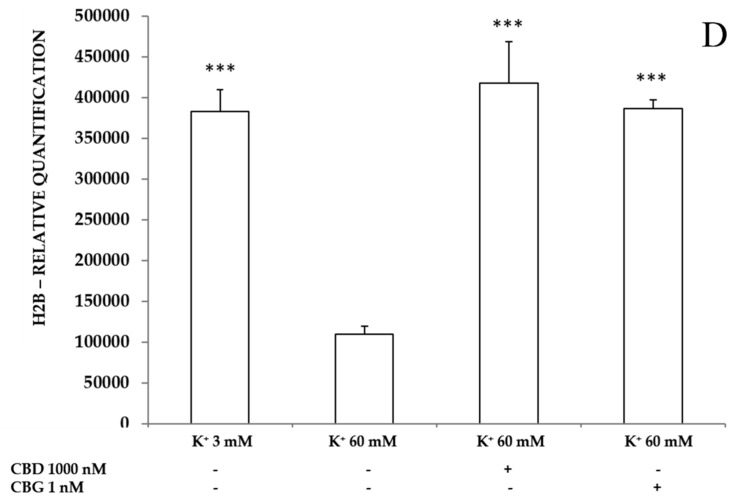
Inhibitory effects of CBD 1000 nM and CBG 1 nM on K^+^ 60 mM-induced reduction in Syt 1 (panel **A**), Stx 1b (panel **B**), CAMK2A (panel **C**) and H2B (panel **D**) levels (expressed as Relative Quantification: R.Q.), in isolated rat cortexes (*n* = 3 for each experimental condition). (Panel **A**): ANOVA, *p* < 0.0001, F = 125.5; *post hoc* test, ***p < 0.001 vs. K^+^ 60 mM group. (Panel **B**): ANOVA, *p* < 0.0001, F = 141.6; *post hoc* test, ***p < 0.001 vs. K^+^ 60 mM group. (Panel **C**): ANOVA, *p* < 0.0001, F = 50.51; *post hoc* test, ** *p* < 0.01 vs. K^+^ 60 mM group. (Panel **D**): ANOVA, *p* < 0.0001, F = 54.27; *post hoc* test, ***p < 0.001 vs. K^+^ 60 mM group.

In the same experimental conditions described in the previous paragraph, an untargeted proteomic analysis was performed. The expression of three proteins involved in exocytosis was found downregulated in presence of the excitotoxic stimulus. The three proteins are synaptotagmin-1 (Syt 1; uniprot code: P21579), syntaxin 1b (Stx 1b; uniprot code: P61268) and calcium/calmodulin-dependent protein kinase type II subunit alpha (CAMK2A; uniprot code: Q9UQM7). The sole CBD 1000 nM showed the capability of blocking the downregulation induced by the K^+^ 60 mM on the expression of the proteins Syt 1, Stx 1b and CAMK2A (Figure 7A–C). 

On the other hand, both CBD and CBG were effective in counteracting the downregulation of histone H2B (H2B: uniprot code: Q99880), induced by the K^+^ 60 mM excitotoxic depolarizing stimulus, restoring its expression to the physiological values (Figure 7D). CBD, but not CBG, proved to be effective in keeping the expression levels of the three proteins involved in exocytosis at values similar to the control sample. Conversely, both terpenophenols were able to blunt the decrease in the expression of histone H2B, a protein involved in DNA replication and repair.

### 2.4. Prediction of Putative Target and Results of Docking Analysis

Through the use of the bioinformatics platform SwissTargetPrediction (http://www.swisstargetprediction.ch/), a pharmacological screening was conducted to identify putative target proteins. Details about the bioinformatic analysis are reported in the Supplementary Materials (Bioinformatic Folder).

Besides well-known interactions with the cannabinoid receptors, the bioinformatics analysis showed potential interactions with a plethora of protein targets that are consistent with a putative multitarget mechanism. In the search for further common targets that could explain the antioxidant and neuroprotective effects induced by both CBD and CBG, the neurokinin 3 receptor (NK3R) was identified as a putative target. The criterion for choosing NK3R was the interaction probability shown by both compounds towards this protein, which was very similar to that observed towards other non-cannabinoid targets (Supplementary Material: Bioinformatics Folder). 

In order to deeply investigate the molecular interactions between the two terpenophenols and the NK3R receptor, a docking study was coupled to the aforementioned bioinformatics prediction. The docking approach permitted us to elucidate the orientation of both CBD and CBG at the active site of NK3R. The related binding affinities, measured as binding free energies (∆G), the inhibition constant (Ki) and the non-bonding interactions were evaluated as well. In Table 3, the affinities of the docked compounds towards the NK3R receptor are shown in comparison with the co-crystalized ligand (retinal), used as a reference compound. Interestingly, the binding affinity of CBG is within an interval of ~ 1.0 kcal/mol of difference from the calculated binding affinity of retinal.

**Figure 8:**
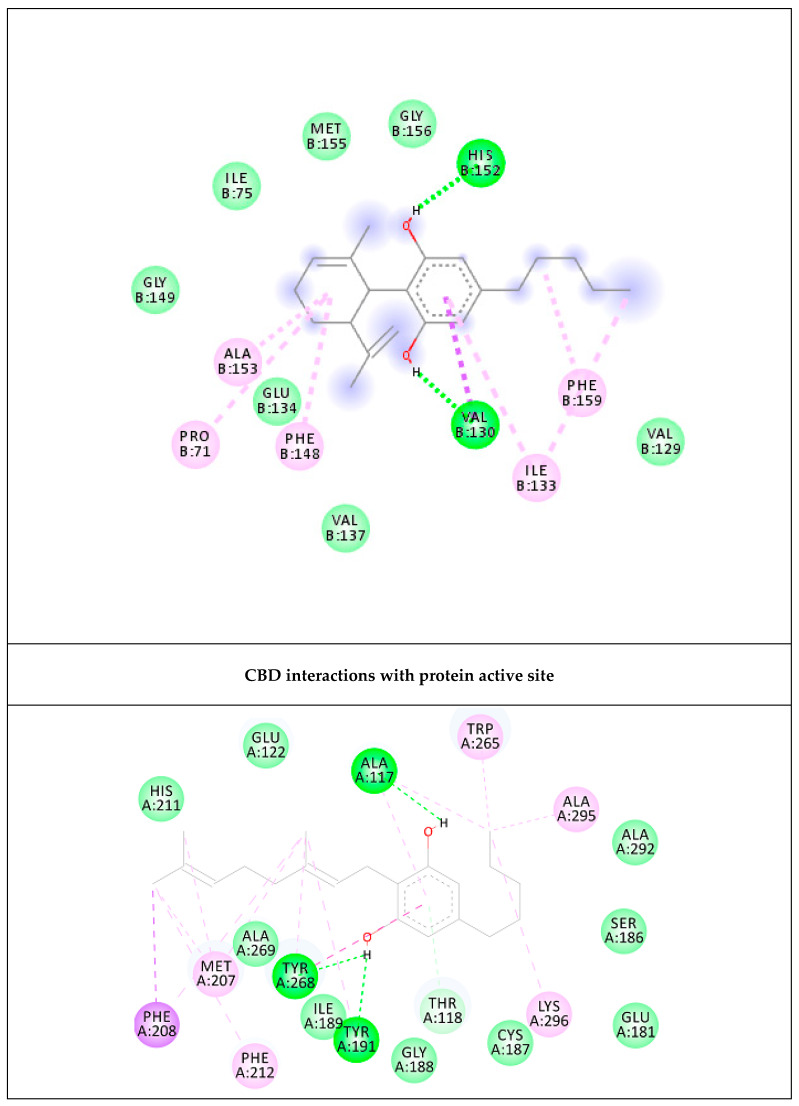

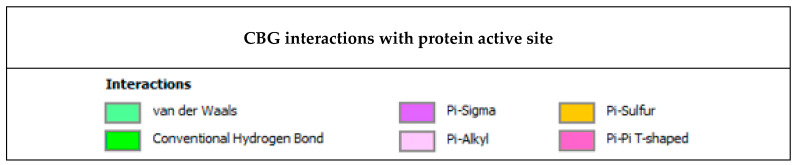
Interactions of the docked compounds, CBD and CBG. The 2D orientations of both docked compounds are shown. The putative interactions with neurokinin 3 receptor (NK3R) are shown with different colors.

**Figure 9:**
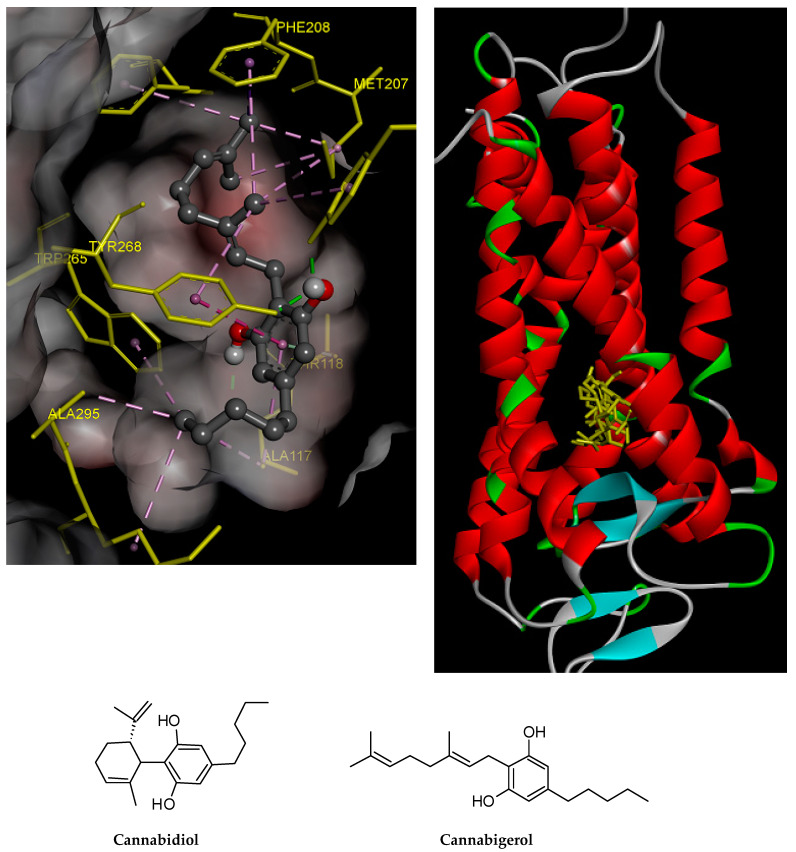
CBD and CBG overlap at the active site of NK3R. The higher affinity of CBG is related to the higher number of hydrogen bonds and pi–pi interactions of CBG with NK3R.

The 2D and 3D orientations of the docked compounds in the active protein site are shown in Figure 8 and Figure 9, respectively. CBG shows higher binding affinity than CBD. This higher affinity may be attributed to the higher number of hydrogen bonds and pi–pi interactions present in the CBG structure in comparison with CBD, as shown in Figure 9.

## 3. Discussion

CBD and CBG were first tested for their intrinsic scavenging/reducing activity. The antiradical effects shown by CBD are, albeit partially, consistent with the literature. This compound is indeed able to interrupt free radical chain reactions and to reduce ROS production through the chelation of transition metal ions involved in the Fenton reaction [9]. CBD’s antiradical effect was found to be comparable with that of the classic antioxidant butylated hydroxytoluene [25], whose efficacy was lower if compared to Trolox, both in DPPH and ABTS assays [26]. Regarding CBG, its intrinsic antiradical activity was unexplored so far, whereas the cannabinoid (CB) receptor-related antioxidant effects described in the scientific literature were studied through the concomitant administration of selective CB1 receptor ligands [27].

Therefore, the two compounds were tested on a cell line of rat astrocytes, and were able to protect them from oxidative stress, reducing ROS production. In agreement with our findings, CBD is known as a potent regulator of oxidative stress, which is a leading cause of neurodegeneration, as it scavenges the reactive oxygen species (ROS) and reduces lipid peroxidation in neuroglia [28,29]. Both in the MTT test and ex vivo model exposed to hydrogen peroxide, however, only the lowest concentration of CBG was effective in counteracting the changes induced by the exposure to hydrogen peroxide. This apparent discrepancy could be due, albeit partially, to the paradoxical and intrinsic pro-oxidant effects of phenolic compounds [30]. The pro-oxidant effect is concentration-dependent and could improve oxidative stress defense systems in cells [31]. This mechanism is particularly important in neurons, which are characterized by a higher susceptibility to oxidative stress, compared to astrocytes. The reason of this difference can be found in the higher rate of oxidative metabolism in neurons compared to glial cells [32]. On the other hand, astrocytes are highly glycolytic cells [32], and their exposure to hydrogen peroxide was found to reduce glycolytic metabolism [33]. It could therefore be hypothesized that the concomitant presence of hydrogen peroxide and high concentrations of CBG could further impair astrocyte metabolism.

Moreover, in another ex vivo model, consisting of rat hypothalamic neurons exposed to hydrogen peroxide, CBG exerted neuroprotection only at low concentrations [11]. These results are consistent with putative pro-oxidant effects occurring at higher concentrations [30]. 

The two compounds CBD and CBG were proven to decrease the apoptosis triggered by the oxidative stress, in line with previously published data demonstrating that cannabinoids are effective in preventing apoptosis in astrocytes [34]. In addition, the modulation of some proteins related to apoptosis occurrence appears consistent with already published papers both for CBG [21] and CBD [35,36]. The latter, in particular, seems able to preserve the involvement of mitochondria by modulating Bax and cleaved caspase expression. These findings appear more relevant if we consider that neurons release damaged axonal mitochondria to adjacent astrocytes for autophagic degradation [37]. Conversely, astrocytes can transfer healthy mitochondria to adjoining neurons for ATP production and recovery from strokes [38].

In the present study, the protective effects of CBD and CBG were investigated also in an ex vivo experimental paradigm, aimed to reproduce the burden of oxidative stress and neurotoxicity occurring in neurodegenerative and neurological diseases. First, isolated rat prefrontal cortexes were challenged with hydrogen peroxide, and the steady state levels of nitrites and the 5-HT concentration were evaluated. The first is an index of nitric oxide (NO) synthesis and nitrosative stress, while the second is a marker of neurotransmitter activity. Both compounds were able to prevent hydrogen peroxide-induced nitrite upregulation and 5-HT downregulation. These results are consistent with various antioxidant mechanisms targeted by both terpenophenols, including the inhibition of inducible NO synthase expression, of lipid peroxidation and of reactive oxygen species production. [27]. The antioxidant effects are also consistent with the prevention of cortex 5-HT depletion. These findings are further supported by the scientific data, addressing natural antioxidants as reliable tools for preventing neurotransmitter activity decline, in aging as well as in neurodegenerative conditions [39]. Furthermore, the protective effects of CBD in the present experimental paradigm is strictly in agreement with data from the literature suggesting the potential application of this terpenophenol in neurodegenerative disease management [40,41]. A potential neuroprotective role for CBG was also reported [42]. In contrast, although the use of CBD in neurological disorders has been extensively studied [7,8,9], there are no studies about CBG. 

Considering the results obtained on hydrogen peroxide-induced 5-HT depletion, both CBD and CBG were tested in another experimental model, where neurotoxicity was induced by 60 mM extracellular levels of K^+^. This paradigm was chosen to mimic the increased 5-HT turnover occurring in many neurological diseases, including migraine, hypoxia/ischemia and epilepsy [23]. Specifically, the excitotoxic depolarizing stimulus is characterized by a supra-physiological neurotransmitter overflow, followed by subsequent stimulated turnover, and paralleled by increased oxidative stress and tissue damage [22,23]. Both terpenophenol compounds prevented serotonin depletion, thus restoring its physiological levels to the values observed in basal (K^+^ 3 mM) and/or physiological depolarizing conditions (K^+^ 15 mM). Nevertheless, sole CBD was recently described to stimulate basal hypothalamic 5-HT levels [11]. This result could be explained with the ability of this compound to restore the physiological values of expression of selected proteins. The three proteins taken into consideration, namely Syt 1, Stx 1b and CAMK2A, are notoriously involved in neurotransmitter exocytosis from ready releasable vesicles and in synaptic plasticity [43,44]. The 5-HT pathway has also been considered as cornerstone to explain the antidepressant effects in mice treated with CBD [45]. Furthermore, the aforementioned finding that CBD stimulated hypothalamic 5-HT levels [11] supports the role of this neurotransmitter in mediating CBD effects. By contrast, CBG appears to be completely ineffective in modulating the expression of the synaptic proteins Syt 1, Stx 1b and CAMK2A. The proteomic analysis also showed a modulation, exerted by both compounds, of histone H2B level, a protein involved in chromosomal stability and DNA replication and repair. Histone activity is modulated by their level of acetylation. Interestingly, the histone deacetylases, a family of enzymes that determine histone level of acetylation, have been reported as targets in neuroprotection [46]. In addition, histone post-transcriptional modifications influence gene expression and play a role in the pathophysiology of neurological diseases, suggesting novel therapeutic approaches [47]. 

In the evaluation of 3-HK and KA levels, the two terpenophenols showed, once again, a different behavior. CBD was able to prevent the upregulation of 3-HK, an index of neurotoxicity, induced by the excitotoxic stimulus. In the same experimental design, CBD reverted the downregulation of KA, whose high expression is a well-known marker of neuroprotection. CBG was instead able to reduce 3-HK and increase KA levels in basal conditions only. Currently, the different effects of CBD and CBG in the cortex could suggest a more selective modulation induced by CBD, compared to CBG, on both 5-HT and kynurenine pathways. These results could indicate the increased 5-HT and KA levels as potential biomarkers of CBD-induced neuroprotection. It cannot be excluded that this difference could depend on discrete receptor mechanisms. It is therefore worth noting the different effect of both compounds on 5-HT receptor (5-HT1A) activity, given that 5-HT1A is activated by CBD and moderately antagonized by CBG [9,10]. On the other hand, in the hydrogen peroxide-induced toxicity model, a decrease in 5-HT release was registered. CBG was here more potent than CBD in blunting neurotransmitter depletion. This finding could be ascribed, albeit partially, to the higher reducing power registered by CBG in the CUPRAC test, compared to CBD.

Taken together, the results obtained in the two experimental models are in line with already published papers demonstrating that decreasing the inflammatory responses of either microglia or astrocytes was shown to be a critical component of the neuroprotective process [48]. Moreover, the intercellular metabolic coordination between neurons and astrocytes can be fundamental in the context of neurodegenerative disorders.

Based on these results, a bioinformatics investigation was conducted in order to identify putative targets responsible for the effects shown by CBD and CBG in the two experimental models. Through the use of a well-recognized online database, the bioinformatics analysis gave a probability for both CBD and CBG to interact with neurokinin 3 B receptor (NK3R), which is expressed both in cortex and neuroglia [49]. This receptor seems to be involved in the pathophysiology of some neurological disorders, such as epilepsy and schizophrenia [50,51]. In the hypothalamus, the neurokinin B is co-expressed with kisspeptin [52], a neuropeptide involved in 5-HT turnover stimulation [53]. Neurokinin B and kisspeptin showed also a cooperative mechanism at the neuroendocrine level [54], whereas the activation of NK3R triggered colon 5-HT release, especially in pro-inflammatory conditions [55]. Interestingly, in our ex vivo model, the cortexes exposed to the neurotoxic stimulus showed a supra-physiological 5-HT overflow and subsequent increase in 5-HT turnover. In this context, it is sensible to hypothesize that the observed efficacy of CBD and CBG in blunting 5-HT turnover could be related, albeit partially, to the NK3R inhibition predicted by the docking analysis. Interestingly, CBG showed a higher affinity against this receptor. It cannot be excluded that this higher affinity could explain the higher potency shown by CBG in counteracting hydrogen peroxide-induced toxicity, both in astrocytes and isolated cortexes. Nevertheless, further pharmacological studies are necessary to confirm the implication of NK3R in the neuroprotection induced by both CBD and CBG.

## 4. Materials and Methods

### 4.1. Drugs

Crystals of CBD and CBG (purity > 99%), were kindly provided by Enecta^®^ Group https://www.enecta.eu. The stock and working solutions (30 mM) were prepared as recently described [11].

### 4.2. Determination of Antioxidant and Enzyme Inhibitory Effects

For the 1,1-diphenyl-2-picrylhydrazyl (DPPH) radical scavenging assay, sample solution was added to 4 mL of a 0.004% methanol solution of DPPH. The sample absorbance was read at 517 nm after a 30 min incubation at room temperature in the dark. DPPH radical scavenging activity was expressed as IC_50_ values.

For the 2,2′-azino-bis(3-ethylbenzothiazoline) 6-sulfonic acid (ABTS) radical scavenging assay, ABTS+ was produced directly by reacting 7 mM ABTS solution with 2.45 mM potassium persulfate and allowing the mixture to stand for 12–16 in the dark at room temperature. Prior to beginning the assay, ABTS solution was diluted with methanol to an absorbance of 0.700 ± 0.02 at 734 nm. Sample solution was added to ABTS solution (2 mL) and mixed. The sample absorbance was read at 734 nm after a 30 min incubation at room temperature. The ABTS radical scavenging activity was expressed as IC_50_ values.

For the cupric ion reducing activity (CUPRAC) activity assay, sample solution was added to premixed reaction mixture containing CuCl2 (1 mL, 10 mM), neocuproine (1 mL, 7.5 mM) and NH4Ac buffer (1 mL, 1 M, pH 7.0). Similarly, a blank was prepared by adding sample solution (0.5 mL) to premixed reaction mixture (3 mL) without CuCl2. Then, the sample and blank absorbances were read at 450 nm after a 30 min incubation at room temperature. The absorbance of the blank was subtracted from that of the sample. CUPRAC activity was expressed as IC50 values.

For the ferric reducing antioxidant power (FRAP) activity assay, sample solution was added to premixed FRAP reagent (2 mL) containing acetate buffer (0.3 M, pH 3.6), 2,4,6-tris(2-pyridyl)-S-triazine (TPTZ) (10 mM) in 40 mM HCl and ferric chloride (20 mM) in a ratio of 10:1:1 (*v*/*v*/*v*). Then, the sample absorbance was read at 593 nm after a 30 min incubation at room temperature. FRAP activity was expressed as IC_50_ values.

For the Cholinesterase (ChE) inhibitory activity assay, sample solution (was mixed with DTNB (5,5-dithio-bis(2-nitrobenzoic) acid, Sigma, St. Louis, MO, USA) (125 µL) and AChE (acetylcholinesterase (Electric ell acetylcholinesterase, Type-VI-S, EC 3.1.1.7, Sigma Aldrich S.r.l., Milan, Italy)), or BChE (butyrylcholinesterase (horse serum butyrylcholinesterase, EC 3.1.1.8, Sigma Aldrich S.r.L.)) solution (25 μL) in Tris–HCl buffer (pH 8.0) in a 96-well microplate and incubated for 15 min at 25 °C. The reaction was then initiated with the addition of acetylthiocholine iodide (ATCI, Sigma Aldrich S.r.l.) or butyrylthiocholine chloride (BTCl, Sigma Aldrich S.r.l.) (25 μL). Similarly, a blank was prepared by adding sample solution to all reaction reagents without enzyme (AChE or BChE) solution. The sample and blank absorbances were read at 405 nm after 10 min incubation at 25 °C. The absorbance of the blank was subtracted from that of the sample and the cholinesterase inhibitory activity was expressed as IC_50_ values. 

Data were represented as the means ± SD of three replications.

### 4.3. In Vitro Studies

#### 4.3.1. Cell Culture and Treatment

The CTX-TNA2 rat astrocytes were purchased from the European Collection of Cell Cultures (ECACC), Sigma-Aldrich, Milan, Italy, and cultured as already indicated [56]. Cells were grown in Dulbecco’s Modified Eagle Medium supplemented with 10% of FBS and penicillin–streptomycin (100 µgmL^−1^) (all from EuroClone SpA Life-Sciences-Division, Milan, Italy) and maintained in a humified atmosphere of 5% CO_2_ at 37 °C. Cells were subcultivated at a ratio of 1.2 to 1.8 and the medium was changed every 3 days. CTX-TNA2 astrocytes were plated in 25 or 75 cm^2^ flasks, according to experimental needing and cells between the eight and the 15 passage were used. 

When indicated, the cells were treated with H_2_O_2_ 300 µM for three hours and different concentrations of CBD (1–1000 nM) and CBG (1–1000 nM). The stock solutions were diluted in cell medium to the point that the concentration of DMSO in the samples was less than 0.0003%. Therefore, the addition of DMSO to the control samples was not necessary. 

#### 4.3.2. MTT Assay

The 3 [4–dimethylthiazol-2yl]-2,5-diphenyl tetrazolium bromide (MTT) growth assay (Sigma-Aldrich S.r.l.) was used to assess cell proliferation as previously described [57]. Briefly, The CTX-TNA2 cells were seeded into 96-well plate at 8 × 10^3^ cells/well and treated as described above. After 24 and 48 h, a medium containing 0.5 mg/mL MTT was added to the cells, and the cells were incubated at 37 °C for 3 h. Following a further incubation of 30 min in DMSO, the absorbance at 570 nm was measured using a Multiscan GO microplate spectrophotometer (Thermo Fisher Scientific, Waltham, MA, USA).

#### 4.3.3. Flow Cytometry Analysis of Reactive Oxygen Species (ROS) Production

Reactive oxygen species (ROS) production was determined as already reported [58] by monitoring by flow cytometry the increase in green fluorescence after labeling the cells (5 × 10^5^) with 5 μmolL^−1^ of 5-(and-6)-chloromethyl-2′,7′-dichlorodihydrofluorescein diacetate, acetyl ester (CM-H_2_DCFDA, Molecular Probes, Invitrogen, Life-Sciences-Division, Milano, Italy) for 40 min followed by 20 min of recovery at 37 °C. The increase in green fluorescence, indicating the production of ROS, was detected by a Cytoflex flow cytometer with an FL1 detector in a log mode (Beckmann Coulter, FL, USA). At least 5000 events for each sample were acquired.

#### 4.3.4. Flow Cytometry Apoptosis Detection

A FITC Annexin-V apoptosis detection kit (BD Pharmingen, San Diego, CA, USA) was used to assess apoptosis, according to the manufacturer’s instructions. Briefly, 10^5^ cells were gently re-suspended in 100 µL of binding buffer and incubated for 15 min at room temperature in the dark with 5 µL of Annexin-V-FITC and 5 µL of Propidium Iodide (PI). After the addition of 200 µL of binding buffer, samples were analyzed with a Cytoflex flow cytometer with the FL1 and FL3 detector in a log mode, using the Cytoexpert analysis software (both from Beckmann Coulter). For each sample, 10,000 events were collected. Viable cells are Annexin-V^neg^/PI^neg^ (unlabelled), necrotic cells are Annexin-V^neg^/PI^pos^, while early and late apoptotic cells are Annexin-V^pos^ and PI^neg^ and PI^pos^, respectively.

#### 4.3.5. Western Blotting Analysis 

The western blowing analysis of Bax and Cyt C was carried out as previously reported [59]. Specifically, CTX-TNA2 lysates (20 µg) were boiled fof 5 min in sample buffer, made of tris-HCl pH 6.8, glycerol, 2-mercaptoethanol, SDS and bromophenol blue. They were then electrophoresed and transferred to nitrocellulose membranes. Nitrocellulose membranes then were blocked in 5% non-fat milk, 10 mmol/L Tris pH7.5, 100 mmol/L NaCl, 0.1 % Tween 20, probed with mouse monoclonal anti-β-tubulin antibody (cat. number T4026, 1:1000; Sigma-Aldrich, St. Louis, MO, USA), mouse monoclonal anti-Bax antibody (cat. number sc-7480, 1:200), mouse monoclonal anti-cytochrome c antibody (cat. number sc-13156, 1:200) (both from Santa Cruz Biotecnologies, Heidelberg, Germany) and incubated in the presence of specific enzyme conjugated IgG horseradish peroxidase. Immunoreactive bands were identified using the ECL detection system (Euroclone, Pero, Milano, Italy) and analyzed by densitometry. Densitometric values were quantified by the CHEMIDOC XRS system and expressed as Integrated Optical Intensity using the QuantiOne 1-D analysis software (Bio-rad Laboratories, Richmond, CA, USA). Values obtained were normalized on densitometric values of internal β-tubulin. Results are expressed as the mean ± SD.

### 4.4. Ex Vivo Studies

#### 4.4.1. Oxidative Stress and Nitrite Production

Sprague–Dawley rats (200–250 g) were sacrificed by CO_2_ inhalation and isolated prefrontal cortex specimens were immediately explanted and exposed to 300 µM H_2_O_2_ and different concentrations of CBD (1–1000 nM) and CBG (1–1000 nM) for one hour, at 37 °C. The nitrite concentration in the resuspension buffer was determined by colorimetric Griess assay. Specifically, cortex specimens were homogenized for 2 min with Potter–Elvehjem homogenizer in 2 mL DMEM buffer. The homogenate was centrifuged at 4500 g for 10 min and nitrite production was determined in the supernatant fraction by mixing 50 µL of the assay buffer with 50 µL of Griess reagent (1.5% sulfanilamide in 1M HCl plus 0.15% N-(1-naphthyl) ethylenediamine dihydrochloride in distilled water, *v*/*v*). After 10 min incubation at room temperature, the absorbance at 540 nm was determined and nitrite concentrations were calculated from a sodium nitrite standard curve. All measurements were performed in triplicate.

#### 4.4.2. A Neurotoxicity Paradigm

Sprague–Dawley rats (200–250 g) were sacrificed by CO_2_ inhalation (Italian Health Ministry authorization N. F4738.N.XTQ, delivered on 11^th^ November 2018) and isolated prefrontal cortex specimens were immediately explanted and incubated in thermostatic shaking bath at 37 °C for 1 h (incubation period), in Krebs–Ringer buffer at the following K^+^ concentrations:

K^+^ 3 mM: basal condition;

K^+^ 15 mM: physiologic depolarizing stimulus;

K^+^ 60 mM: excitotoxic depolarizing stimulus.

This paradigm was designed according to previous ex vivo studies [22,23], which demonstrated the capability of freshly explanted brain tissue to still synthesize and release neurotransmitters and respond to pro-oxidant and anti-oxidant stimuli, after an incubation period at 37 °C and in artificial cerebrospinal fluid (Krebs–Ringer buffer) that mimics the physiological conditions of neurons in vivo. Cortexes were stimulated with CBD (1000 nM) and CBD (1 nM). Afterwards, specimens were homogenized in perchloric acid solution (0.05 M) for the extraction and quantification of nitrites, via the aforementioned colorimetric Griess assay, and serotonin (5-HT), 5-hydroxyindoleacetic (5HIIA),3-hydroxykinurenine (3-HK) and kinurenic acid (KA) (ng/mg wet tissue) via high performance liquid chromatography analysis. Additionally, an untargeted proteomic analysis was carried out on the cortex homogenate. The analytical protocols for 5-HT, 5HIIA, 3-HK, KA and protein level determinations are described below. The analyses were conducted in triplicate and in independent facilities with blind modality.

#### 4.4.3. High Performance Liquid Chromatography (HPLC) Determination of Serotonin (5-HT), 5-Hydroxyindoleacetic (5HIIA) and 3-Hydroxykinurenine (3-HK)

Tissue 3-HK, 5-HT and 5HIIA levels were analyzed through an HPLC apparatus consisting of a Jasco (Tokyo, Japan) PU-2080 chromatographic pump and an ESA (Chelmsford, MA, USA) Coulochem III coulometric detector, equipped with a microdialysis cell (ESA-5014b), a porous graphite working electrode and a solid state palladium reference electrode. The analytical conditions for identification and quantification were selected according to previous studies [11]. Specifically, the analytical cell was set at −0.150 V for detector 1 and at +0.300 V for detector 2, with a range of 100 nA. The chromatograms were monitored at the analytical detector 2. Integration was performed by Jasco Borwin Chromatography software version 1.5. The chromatographic separation was performed by isocratic elution on a Phenomenex Kinetex reverse phase column (C18, 150 × 4.6 mm i.d., 2.6 µm). Regarding the separation of 5HIIA and 5-HT, the mobile phase was (10:90, *v/v*) acetonitrile and 75 mM pH 3.00 phosphate buffer containing octanesulfonic acid 1.8 mM, EDTA 30 µM and triethylamine 0.015% *v/v*. The mobile phase for 3-HK analysis consisted of 1.5% acetonitrile, 0.9% triethylamine, 0.59% phosphoric acid, 0.27 mM EDTA, and 8.9 mM octanesulfonic acid. The flow rate was 0.6 mL/min and the samples were manually injected through a 20 µL loop. Analyte peaks were identified by comparison with the retention time of pure standard. Sample concentrations were calculated by linear regression curve (y = bx + m) obtained with standard. Neither internal nor external standards were necessary for analyte quantification in the cortex homogenate, and all tests performed for method validation and yielded results in accordance with limits indicated in official guidelines for applicability in laboratory trials. The standard stock solutions of 5HIIA, 5-HT and 3-HKat 2 mg/mL were prepared in bidistilled water containing 0.004% EDTA and 0.010% sodium bisulfite. The stock solutions were stored at 4 °C. Work solutions (1.25–20.00 ng/mL) were obtained daily by progressively diluting the stock solutions in the mobile phase.

#### 4.4.4. HPLC–Fluorimetric Determination of Kinurenic Acid (KA)

The KA quantitative determination was performed by using a liquid chromatograph (MOD. 1525, Waters Corporation, Milford MA, USA) equipped with a fluorimetric detector (MOD. 2475, Waters Corporation), a C18 reversed-phase column (AcclaimTM 120, 3 µm, 2.1 × 100 mm, Dionex Corporation, Sunnyvale, CA, USA), and an online degasser (Biotech 4-CH degasi compact, LabService, Anzola Emilia, Italy). The separation was conducted in isocratic conditions and the mobile phase consisted of 250 mM zinc acetate, 50 mM sodium acetate, and 3% acetonitrile (pH adjusted to 6.2 with glacial acetic acid), using a flow rate of 1.0 mL/min. In the eluate, the KA was identified and measured fluorimetrically (excitation: 344 nm; emission: 398 nm).

#### 4.4.5. Proteomic Analysis

After protein quantification, a volume corresponding to 50 μg of proteins was loaded onto a Nanosep 10-kDa-cutoff filter (Pall Corporation, Michigan, USA) and digested according to the protocol we routinely use in our laboratory, adapted from Distler et al. Briefly, the sample was washed twice with 200 μL urea buffer (8M urea, 100 mM Tris pH 8.5 in milliQ water) to remove the detergents present in the lysis buffer. The proteins on the filter where subsequently reduced and alkylated by adding 100 μL of DTT solution (8 mM Dithiothreitol in urea buffer) and 100 μL of IAA solution (50 mM Iodoacetamide in urea buffer). For protein digestion, the buffer was exchanged with 50 mM ammonium bicarbonate, before adding trypsin to a ratio of 1:50 (enzyme:substrate). The reaction was incubated for 16 h at 37 °C, and the mixture of peptides was collected by centrifugation, acidified with 10% trifluoroacetic acid and stored at −20°C until analysis. Each digested protein sample was analyzed in technical triplicate by LC-MS/MS using a Proxeon EASY-nLCII (Thermo Fisher Scientific, Milan, Italy) chromatographic system coupled to a Maxis HD UHR-TOF (Bruker Daltonics GmbH, Bremen, Germany) mass spectrometer. Peptides were loaded on the EASY-Column C18 trapping column (2 cm L., 100 µm I.D, 5 µm ps, Thermo Fisher Scientific), and subsequently separated on an Acclaim PepMap100 C18 (75 µm I.D., 25 cm L, 5 µm ps, Thermo Fisher Scientific) nano scale chromatographic column. The flow rate was set to 300 nL/min and the gradient was from 3% to 35% of B in 80’ followed by 35% to 45% in 10’ and from 45% to 90% in 11’. Mobile phase A was 0.1% formic acid in H_2_O and mobile phase B was 0.1% formic acid in acetonitrile. The mass spectrometer, typically providing 60,000 FMHW resolution throughout the mass range, was equipped with a nano ESI spray source. The mass spectrometer was operated in positive ion polarity and auto MS/MS mode (Data Dependent Acquisition (DDA)), using N2 as collision gas for CID fragmentation. Precursors in the range 350 to 2200 *m*/*z* (excluding 1220.0–1224.5 *m*/*z*) with a preferred charge state +2 to +5 (excluding singly charged ions) and absolute intensity above 4706 counts were selected for fragmentation in a maximum cycle time of 3 s. After acquiring one MS/MS spectrum, the precursors were actively excluded from selection for 30 s. Isolation width and collision energy for MS/MS fragmentation were set according to the mass and charge state of the precursor ions (from 3 to 9 Da and from 21 eV to 55 eV). The in-source reference lock mass (1221.9906 *m*/*z*) was acquired online throughout the runs. Raw data were processed using PEAKS Studio v7.5 software (Bioinformatic Solutions Inc, Waterloo, Canada) using the ‘correct precursor only’ option. The mass lists were searched against a human database downloaded from the UniProt website (https://www.uniprot.org/) to which a list of common contaminants was appended (as of June 2017; 20,441 entries). The carbamidomethylation of cysteines was selected as fixed modification and oxidation of methionines and the deamidation of asparagine and glutamine were set as variable modifications. Non-specific cleavage was allowed at one end of the peptides, with a maximum of 2 missed cleavages. Values of 10 ppm and 0.05 Da were set as the highest error mass tolerances for precursors and fragments, respectively. An expression analysis for the abundance of identified proteins was performed through the label-free quantification module PEAKS-Q, part of PEAKS Studio v7.5. This quantification method is based on the relative areas of the extracted ion chromatograms of peptides detected in multiple samples and applies the expectation–maximization algorithm to detect and resolve overlapping features. The features of the same peptide from different samples are aligned using a high-performance retention time alignment algorithm. 

### 4.5. Bioinformatic and Docking Studies

#### 4.5.1. Bioinformatic Analysis: Prediction of Putative Target Proteins

Chemical structures were prepared and converted in canonical SMILES using ChemSketch software. The SMILES were then processed by the SwissTargetPrediction (http://www.swisstargetprediction.ch/) platform, for predicting putative protein targets. The name of identified targets was normalized according to the UniProt database (https://www.uniprot.org/).

#### 4.5.2. Docking Calculations

Two compounds, namely, CBD and CBG were selected to study their binding affinity towards rhodopsin protein using docking calculation. The routine steps for docking calculations involve the preparation of the inhibitors and the protein. The crystal structure of the protein was downloaded from Protein Data Bank (PDB), PDB code: 1F88. In order to prepare the protein for docking calculations, all water molecules and co-crystalized compounds were removed. This step was followed by adding polar hydrogen atoms and neutralized using Autodock4 program (Molinspiration Database). The starting structures of CBD and CBG were optimized to their ground state structures using the AM1 semiempirical method and the 3D structures were saved in mol2 format. The protein was immersed in a 3D grid box with 60 × 60 × 60 dimensions with 0.375 Å distance between points. Lamarckian genetic algorithm was used to calculate the docking free energy of 250 conformations for each inhibitor. The docking results were clustered and organized according to the docking free energy. The binding site was localized and the non-bonding interactions were elucidated using Discovery Studio 5.0 visualizer.

### 4.6. Statistical Analysis

As for the in vitro test analysis, bio-pharmacological data, expressed as means ± S.D., and scavenging/reducing and enzyme inhibitory assays were analyzed by an analysis of variance (ANOVA) coupled with Tukey’s post-hoc test. Statistical significance was set at *p* < 0.05, and GraphPad Prism version 5.01 for Windows (GraphPad Software, San Diego, CA) was employed for the analysis. The statistical analysis to calculate the total number of animals (*n* = 19) used in the two ex vivo paradigms was performed with the software G*Power (v3.1.9.4). Study potency (1−β) and significance level (α) were 0.8 and 0.05, respectively. 

## 5. Conclusions

The present study compared the antioxidant and neuroprotective effects of CBD with those of CBG. The protection exerted by the two compounds on the astrocyte cell line suggests that the treatment of oxidative conditions should also take into consideration the targeting of non-neuronal brain cells. In the ex vivo model, CBG, besides preventing 5-HT depletion induced by different stimuli, was less effective than CBD in restoring the levels of proteins involved in neurotransmitter exocytosis. On the other hand, CBG was effective at lower concentrations than CBD, especially in rat astrocytes and isolated cortexes challenged with the pro-oxidant stimulus constituted by hydrogen peroxide. Bioinformatics and docking analyses predicted inhibitory effects induced by these compounds toward the NK3R receptor that, besides corroborating the observed bio-pharmacological data, suggest further investigations about the roles of CBD and CBG as neuroprotective agents. 

## Figures and Tables

**Table 1:** Anti-cholinesterase activity.

	AChE Inhibition	BChE Inhibition
CBD	1.04 ± 0.02 ^a^	1.88 ± 0.11 ^a^
CBG	1.15 ± 0.03 ^b^	1.48 ± 0.11 ^b^
Galantamine	0.01 ± 0.001 ^c^	0.02 ± 0.002 ^c^

IC_50_ values (mM) are reported as mean ± S.D. of three parallel experiments (*n* = 3). Galantamine represents the reference AChE and BChE inhibitor. Different letters indicate significant differences in the samples (*p* < 0.05).

**Table 2:** Scavenging/Reducing activity.

	DPPH Scavenging Ability Test	ABTS Scavenging Ability Test	Ferric Reducing Antioxidant Power (FRAP)	Cupric Reducing Antioxidant Capacity (CUPRAC)
CBD	1.87 ± 0.04 ^b^	1.04 ± 0.01 ^a^	>2	0.94 ± 0.01 ^a^
CBG	1.95 ± 0.01 ^a^	1.03 ± 0.01 ^b^	>2	0.59 ± 0.01 ^b^
Trolox	0.21 ± 0.01 ^c^	0.41 ± 0.01 ^c^	0.18 ± 0.01	0.50 ± 0.01 ^c^

IC_50_ values (mM) are reported as mean ± S.D. of three parallel experiments (*n* = 3). Trolox is used as reference radical scavenger/reducing agents. Different letters indicate significant differences in the samples (*p* < 0.05).

**Table 3:** The calculated binding free energy, ∆G, in kcal/mol, the inhibition constant Ki, the key residues and the number of hydrogen atoms of the docked compounds.

Targets	∆G (K_i_)	Key Residues	No. of HB
CBD	−6.72 (12.04 µM)	His152 (HB), Val130 (HB), Ala153, Ile133, Pro71, Phe159, Phe148	2
CBG	−10.36 (25.5 nM)	Tyr191 (HB), Ala117 (HB), Tyr268 (HB & pi–pi), Trp265, Ala295, Lys296, Phe212, Met207, Phe208	3
Control RET	−10.81 (11.8 nM)		

## Supplementary Materials

Supplementary materials can be found at https://www.mdpi.com/1422-0067/21/10/3575/s1.
