# Supplementary material for: Antioxidant and Neuroprotective Effects Induced by Cannabidiol and Cannabigerol in Rat CTX-TNA2 Astrocytes and Isolated Cortexes
Source: Int J Mol Sci. 2020 May 18;21(10):3575. doi: 10.3390/ijms21103575 (PMC7279038; doi:10.3390/ijms21103575)
Supplement: Supplementary file 1 [file ijms-21-03575-s001.zip › ijms-796889-supplementary/Supplementary Material/Supplementary Proteomic Analysis.docx]

*Protein extraction and Filter-aided sample preparation*

After protein quantification, a volume corresponding to 50 ug of proteins was loaded onto a Nanosep 10-kDa-cutoff filter (Pall Corporation – Michigan USA) and digested according to the protocol we routinely use in our laboratory, adapted from Distler et al. Briefly the sample was washed twice with 200 uL Urea buffer (8M Urea, 100 mM Tris pH 8.5 in milliQ water) to remove the detergents present in the lysis buffer. The proteins on the filter where subsequently reduced and alkylated by adding 100 uL of DTT solution (8 mM Dithiothreitol in Urea buffer) and 100 uL of IAA solution (50 mM Iodoacetamide in Urea buffer). For protein digestion, the buffer was exchanged with 50 mM Ammonium Bicarbonate, before adding trypsin to a ratio of 1:50 (enzyme:substrate). The reaction was incubated for 16 h at 37 °C, and the mixture of peptides was collected by centrifugation, acidified with 10% trifluoroacetic acid and stored at -20°C until analysis.

*LC-MS/MS label free shotgun proteomics*

Each digested protein sample was analysed in technical triplicate by LC-MS/MS using a Proxeon EASY-nLCII (Thermo Fisher Scientific, Milan, Italy) chromatographic system coupled to a Maxis HD UHR-TOF (Bruker Daltonics GmbH, Bremen, Germany) mass spectrometer. Peptides were loaded on the EASY-Column C18 trapping column (2 cm L., 100 µm I.D, 5 µm ps, Thermo Fisher Scientific), and subsequently separated on an Acclaim PepMap100 C18 (75 µm I.D., 25 cm L, 5 µm ps, Thermo Fisher Scientific) nano scale chromatographic column. The flow rate was set to 300 nL/min and the gradient was from 3 to 35% of B in 80’ followed by 35 to 45% in 10’ and from 45 to 90% in 11’. Mobile phase A was 0.1% formic acid in H2O and mobile phase B was 0.1% formic acid in acetonitrile. The mass spectrometer, typically providing 60,000 FMHW resolution throughout the mass range, was equipped with a nanoESI spray source. The mass spectrometer was operated in positive ion polarity and Auto MS/MS mode (Data Dependent Acquisition - DDA), using N2 as collision gas for CID fragmentation. Precursors in the range 350 to 2,200 m/z (excluding 1,220.0–1,224.5 m/z) with a preferred charge state +2 to +5 (excluding singly charged ions) and absolute intensity above 4,706 counts were selected for fragmentation in a maximum cycle time of 3 seconds. After acquiring one MS/MS spectrum, the precursors were actively excluded from selection for 30 seconds. Isolation width and collision energy for MS/MS fragmentation were set according to the mass and charge state of the precursor ions (from 3 to 9 Da and from 21 eV to 55 eV). In-source reference lock mass (1,221.9906 m/z) was acquired online throughout the runs.

*Bioinformatics Processing*

Raw data were processed using PEAKS Studio v7.5 software (Bioinformatic Solutions Inc, Waterloo, Canada) using the ‘correct precursor only’ option. The mass lists were searched against human database downloaded from the UniProt website (https://www.uniprot.org/) to which a list of common contaminants was appended (as of June 2017; 20,441 entries). Carbamidomethylation of cysteines was selected as fixed modification and oxidation of methionines and deamidation of asparagine and glutamine were set as variable modifications. Non-specific cleavage was allowed to one end of the peptides, with a maximum of 2 missed cleavages. 10 ppm and 0.05 Da were set as the highest error mass tolerances for precursors and fragments respectively. Expression analysis for abundance of identified proteins was performed through the label-free quantification module PEAKS-Q, part of PEAKS Studio v7.5. This quantification method is based on the relative areas of the extracted ion chromatograms of peptides detected in multiple samples and applies the expectation-maximization algorithm to detect and resolve overlapping features. The features of the same peptide from different samples are aligned using a high-performance retention time alignment algorithm.

Gene Ontology and pathway analysis were performed with QIAGEN’s Ingenuity Pathway Analysis (IPA, QIAGEN Redwood City, www.qiagen.com/ingenuity, Build version: 321501M Content version: 21249400). The analysis was performed using the core analysis option, considering direct and indirect relationships among genes, Confidence Filter for microRNAs set to experimentally observed or predicted with high probability.
